# Training of oncologists: results of a global survey

**DOI:** 10.3332/ecancer.2020.1074

**Published:** 2020-07-17

**Authors:** Divyanshi Jalan, Fidel Rubagumya, Wilma M Hopman, Verna Vanderpuye, Gilberto Lopes, Bostjan Seruga, Christopher M Booth, Scott Berry, Nazik Hammad

**Affiliations:** 1 McMaster University, Hamilton ON L8S 4L8, Canada; 2 Rwanda Military Hospital, Rwanda 0000; 3 Kingston General Hospital Research Institute, Kingston, Ontario K7L 2V7, Canada; 4 Korle Bu Teaching Hospital, Accra, Ghana 0000; 5 University of Miami and Sylvester Comprehensive Cancer Center, Miami, FL 33136, USA; 6 Division of Medical Oncology, Institute of Oncology Ljubljana, Ljubljana, Slovenia 1000; 7 Department of Oncology, Queen's University, Kingston ON K7L 5P9, Canada; 8 Department of Oncology, University of Toronto, Toronto ON M4N 3M5, Canada

**Keywords:** education, medical, global health, medical oncology, radiation oncology, teaching

## Abstract

While several studies have highlighted the global shortages of oncologists and their workload, few have studied the characteristics of current oncology training. In this study, an online survey was distributed through a snowball method for cancer care providing physicians in 57 countries. Countries were classified into low- or lower-middle-income countries (LMICs), upper-middle-income countries (UMICs) and high-income countries (HICs) based on World Bank criteria. A total of 273 physicians who were trained in 57 different countries responded to the survey: 33% (90/273), 32% (87/273) and 35% (96/273) in LMICs, UMICs and HICs, respectively. About 60% of respondents were practising physicians and 40% were in training. The proportion of responding trainees was higher in LMICs (51%; 45/89) and UMICs (42%; 37/84), than HICs (19%; 28/96; *p* = 0.013). A higher proportion of respondents from LMICs (37%; 27/73) self-fund their core oncology training compared to UMICs (13%; 10/77) and HICs (11%; 10/89; *p* < 0.001). Respondents from HICs were more likely to complete an accepted abstract, poster and publication from their research activities compared to respondents from UMICs and LMICs. Respondents identified several barriers to effective training, including skewed service to education ratio and burnout. With regard to preparedness for practice, mean scores on a 5-point Likert scale were low for professional tasks like supervision and mentoring of trainees, leadership and effective management of an oncology practice and understanding of healthcare systems irrespective of country grouping. In conclusion, the investment in training by the public sector is vital to decreasing the prevalence of self-funding in LMICs. Gaps in research training and enhancement of competencies in research dissemination in LMICs require attention. The instruction on cancer care systems and leadership needs to be incorporated in training curricula in all countries.

## Introduction

Health systems worldwide face diverse challenges related to a global health workforce crisis [1]. There is not only a shortage in the number of healthcare workers but also disparities among different geographic regions. The World Health Organization (WHO) has advocated for efforts to align the education of healthcare professionals to the health needs of the community [1]. There are several barriers to providing high-quality professional education, particularly in low- and lower-middle-income countries (LMICs), ranging from insufficient basic infrastructure and equipment to shortage of teaching staff [2].

The complexity of cancer care poses unique challenges to health systems worldwide. In 2013, there were an estimated 14.9 million new cases of cancer worldwide and 8.2 million deaths [3]. This burden is increasing with a 45% projected increase in global cancer deaths by 2030 estimated by the WHO. This trend poses a particular threat in LMICs that are ill-equipped to deal with complex and expensive cancer treatments [3, 4]. Although scaling up educational programs to produce more healthcare professionals is beneficial, these efforts need to be accompanied by educational reforms that will provide graduates with the necessary competencies [1]. It is not uncommon for the patients in LMICs with potentially curable disease to receive sub-optimal radiotherapy and systemic therapy [5].

Globally, becoming an oncologist can involve various pathways following medical school. Core oncology training can take the form of medical oncology, radiation oncology, clinical oncology and surgical oncology. Clinical oncologists are trained to use both radiotherapy and systemic therapy for the treatment of malignant disease [6]. Clinical oncologists are becoming uncommon in high-income countries (HICs) (except for the UK) but remain the main providers of cancer care in many LMICs [7]. Due to the rapid expansion of the knowledge in both medical and radiation oncology in the past two decades [8], simultaneously attaining adequate competency in both of these specialities may not be feasible [5].

While several studies have highlighted the global shortages of oncologists and their workload [9, 10], few have studied the characteristics of current oncology training [11, 12]. We are not aware of any study that explores this issue from a global perspective or includes insights from LMICs. To address this gap in knowledge, we surveyed oncologists and trainees globally to gain an understanding of their training, and its relevance in preparing them for their current practice.

## Methods

### Study participants

Medical oncologists, radiation oncologists, clinical oncologists and other specialists such as surgeons and family physicians who provide systemic therapy or radiotherapy from 57 countries were invited to complete an email-distributed self-administered survey. These physicians were identified using pre-existing regional oncology databases, where possible, through a network of contacts described previously [10]. This study was approved by the Research Ethics Board of Queen’s University.

### Survey design and distribution

The 71-question survey comprised five sections: 1. demographics, 2. description of the physician/trainee’s current or future practice/setting, 3. description of their medical, core oncology and fellowship training, 4. the relevance of their training in preparing them for their current or future practice and 5. future directions in oncology. The survey was designed by the study investigators primarily working in HICs and subsequently revised based on the feedback from study investigators who practised in diverse environments. The feedback was also sought from trainees. The final revised survey was only distributed in English and took approximately 15 minutes to complete.

An email invitation detailing the purpose of the study and a hyperlink to the survey were sent via Qualtrics, an online survey software, to a regional contact, to subsequently distribute to their regional membership/network. The consent for the survey was detailed in the opening email invitation and submission of survey was deemed provision of consent. The survey was distributed in January 2018, and email reminders were sent in March and May 2018.

### Data analysis

We classified the countries, where respondents completed their core oncology training, into LMICs, upper-middle-income countries (UMICs) and HICs on the basis of World Bank criteria [13]. Our study aimed to describe oncologists’ preparedness for practice based on their training across LMICs, UMICs and HICs. Data were initially collected through Qualtrics, and then exported to IBM Statistical Package for the Social Sciences (SPSS) for Windows (version 24.0, Armonk New York, 2018). We excluded data from respondents who only completed the demographics section of the survey. Proportions for responses were reported with the number of people who responded to a particular question as the denominator. Pearson χ**^2^** tests were used to test for the difference in proportions, and the Kruskal–Wallis test was used to compare ordinal and continuous data by income stratification. We used thematic analysis for free-text responses.

## Results

We received a total of 334 responses to the survey. We excluded data from 46 respondents (14%) who only completed the demographics section or less. Of the remaining 288, 15 (5%) did not provide information on where they completed their core oncology training and were also excluded from further analysis. Overall, 273 physicians practising in 57 different countries, 33% (90/273), 32% (87/273) and 35% (96/273) in LMICs, UMICs and HICs, respectively, were included in our study. All continents were represented with 49 (18%) from Africa, 61 (22%) from Asia, 55 (20%) from Europe, 61 (22%) from North America and 47 (17%) from South America.

### Characteristics of study participants and current practice

The median age of respondents was 40 years, and 46% (125/271) were female participants (Table 1). The proportion of female respondents was significantly higher in HICs as compared to UMICs and LMICs. At the time of survey completion, 60% of respondents were practising physicians and 40% were in training. The proportion of trainees among the respondents was higher in LMICs (51%; 45/89) and UMICs (42%; 37/84), as compared to HICs (19%; 28/96; *p* = 0.013).

Of all respondents, 71% (195/273) provide only systemic therapy, 5% (14/273) provide only radiation therapy and 23% (64/273) provide both types of therapy. Respondents from LMICs were more likely to provide both systemic therapy and radiation therapy as compared to UMICs and HICs.

### Characteristics of core oncology training program

Overall, the median duration of respondents’ core oncology training program was 4 years, with no significant differences across the three economic groups (Table 2). Overall, 67% (161/240) of the respondents reported undertaking medical oncology in their core oncology training program, and 3% (8/240) reported having no formal core oncology training at all. Respondents from LMICs were more likely to have had their core oncology training in clinical oncology as compared to UMICs and HICs (*p* <0 .001).

Of those respondents who were practising at the time of survey completion, 19% (38/196) overall, reported completing their core oncology training in a different country than the one they currently practice in. Obtaining core oncology training in a different country occurred more frequently for respondents from LMICs (33%; 16/49) compared to UMICs (18%; 12/66) and HICs (12%;10/81; *p* < 0.001). However, most respondents remained in a country with a similar standing as per the World Bank criteria: 63% of respondents (10/16) who completed their training in LMIC, 83% of respondents (10/12) from UMIC and 80% of respondents (8/10) from HIC, currently practice in LMIC, UMIC and HIC, respectively.

Funding for core oncology training programs reportedly come from a variety of sources, such as the associated hospital, affiliated university, self-funding from the trainees, scholarships or funding from the host country, international organizations and other sources (Table 2). A higher proportion of respondents from LMICs (37%; 27/73) are reportedly self-funded as compared to UMICs (13%; 10/77) and HICs (11%; 10/89; *p* < 0.001).

Although respondents across all three economic groups reported undertaking a research project during their core oncology training, there were significant differences in the outcomes of the research. Respondents from HICs were more likely to report completing an accepted abstract, poster and publication from their research activities as compared to respondents from UMICs and LMICs (abstract: 37/72 (51%) from HICs, 18/66 (27%) from UMICs, 24/65 (37%) from LMICs, *p* = 0.014; poster: (42/72 (58%) from HICs, 28/66 (42%) from UMICs, 13/65 (20%) from LMICs, *p* < 0.001; publication: 43/72 (60%) from HICs, 32/66 (49%) from UMICs, 24/65 (37%) from LMICs, *p* = 0.029)). There were no significant differences in the reported oral presentations between the three economic groups.

### Preparedness for practice

Respondents were asked to report how well their training had prepared them in 15 professional tasks that included clinical competencies in addition to advocacy, leadership and system thinking. Overall, mean scores were lower for all of the non-medical expert roles (Table 3). When responses were analysed across LMICs, UMICs and HICs, there were significant differences for ten of the 15 professional tasks, with highest levels of preparedness reported by respondents from HICs. Respondents from HICs reportedly felt the least prepared as compared to LMICs and UMICs in terms of engagement in activities that raise awareness about advancements in oncology to a wider audience (*p* = 0.017). When analysed across the three groups, there were no significant differences in overall preparedness for practice (*p* = 0.416).

### Challenges in core oncology training

Respondents were asked to comment on the most pressing challenges faced during their training. Overall, 34% (92/273) of respondents provided free-text answers for this question with comparable representation from all economic regions (38% (35/92) from LMIC, 28% (26/92) from UMIC and 34% (31/92) from HIC). We received free-text answers from 39% (35/90), 30% (26/87) and 32% (31/96) of respondents from LMIC, UMIC and HIC, respectively.

Using thematic analysis, the following themes were identified as challenges faced by trainees: 1) busy clinical practice, 2) lack of research opportunities, 3) lack of supervision and mentorship, 4) burnout and 5) lack of leadership training.

Overall, several respondents reported a high volume of patients, ‘pressures of service delivery’, an extremely busy work schedule with ‘100+ hours of work in a week’, and a clinical workload that ‘is becoming crushing, unmanageable, and unfulfilling in more and more ways’. One respondent reported that the ‘burden of clinical activity leaves very small room for academic activities’.

Although respondents expressed interest in conducting research, and felt pressured ‘to be considered a productive resident’, they were limited in what they could accomplish due to time constraints as well as lack of mentorship and guidance on how to pursue a research project and how to be research oriented. Specifically, respondents from LMICs and UMICs raised concerns about lack of clinical trials at their training sites and lack of funding, whereas time constraint due to high clinical burden was a universal challenge.

A large number of respondents from LMICs compared to UMICs and HICs also reported a lack of structured teaching and minimal supervision from staff in the clinical setting, with ‘pressing patient volume that limits the available trainers’ and ‘lack of qualified trainers’. One respondent felt that trainees should be provided with a solid foundation upon which to build their skills, rather than being ‘thrown in the swimming pool to then do [their] best to learn how to swim’. Another respondent from an LMIC identified that they were ‘not properly inducted into the program’ and that ‘there is no facilitation in the wards’.

Burnout, and lack of attention towards work-life balance was another common theme, with ‘limited resources identified to deal with work-related fatigue and burnout’ for both trainees and staff. Due to the high demand for a service commitment, one respondent’s ‘trainer felt that [they] should be able to “get on” and manage difficult situations’. Another respondent considered leaving their clinical activities during training, but ‘fortunately succeeded in overcoming this difficult period [during their] first few years as a medical oncologist’.

Lack of leadership training was another recurring theme, predominantly in responses from trainees in HICs. One respondent felt that ‘understanding management of resources and policy was the weakest area of [their] training’. Respondents from HICs reported a need for more leadership training, ‘how to optimise the health care system [they] are working in’ and ‘to consider administrative/leadership skills as trainable skills rather than purely based on experience’.

For those providing radiation therapy, lack of infrastructure and technology was a significant challenge, including machinery that ‘was not working during the training and patients were referred abroad for radiation treatment’. One respondent mentioned that foundational skills are lacking as they ‘do not have proper lectures on critical subjects like radiology and radiotherapy delivery, including basic and applied physics’.

### Clinical oncology and future trends in training

A higher proportion of respondents from HICs (77%; 56/73) and UMICs (79%; 67/85) as compared to LMICs (29%; 20/69; *p* < 0.001) reported that medical oncology and radiation oncology are separate in their country of practice. Respondents from HICs (62%; 48/77) and UMICs (56%; 37/66) were more likely to wish for separation of training into medical oncology and radiation oncology as compared to LMICs (36%; 25/69; *p* = 0.020). Of those respondents from LMICs who do have clinical oncology in their country of practice, 75% (37/49) envisioned that clinical oncology would be completely separated into medical and radiation oncology within the next 20 years.

## Discussion

Our study provides perspectives from practising and in-training oncologists regarding the current state of oncology training worldwide. Overall, there is representation from 57 different countries and equivalent representation from each of the three economic groups of countries.

It is concerning that a higher proportion of respondents from LMICs reported being self-funded for their core oncology training as compared to UMICs and HICs. Self-funding has future implications for workforce selection and may increase disparities within and between countries by adversely affecting the most disadvantaged populations and diminishing the social accountability of training programs. Professional education has been overlooked in many LMICs. The neglect is to some extent understandable in view of the fact that professional education is expensive, time-consuming and often not entirely attuned to the local disease burden. However, professionals offer the human link for translation of knowledge-related global public goods to the requirements of local realities [2]. As such, investment in training will help LMICs meet the sustainable development goals for non-communicable diseases by 2030 and beyond. Public financing is the most important source of sustainable funding in all the countries, poor or rich. Such investments should be allocated to develop a skill mix that is appropriate to national contexts [2]. Our survey did not explore the nature of self-funding and its impact on career choices. However, in the HIC setting, American oncology fellows with greater education debts were more likely to pursue private practices and less likely to plan an academic career [14]. Although respondents from all three groups reported undertaking research during their training, a lower proportion of respondents from LMIC reported being able to publish their research in the form of an abstract, poster or publication. Providing quality cancer care requires familiarity with current research and evidence-based guidelines. In fact, Are *et al* [15] recently demonstrated that increasing national rates of cancer-related research activity correlated with a decrease in cancer-specific mortality. Despite infrastructure challenges, it is reassuring that many oncologists from LMICs engage in research during their oncology training. Challenges in the dissemination of this research in terms of abstract and manuscript publishing could be related to lack of skill and the knowledge of the scientific publishing process and other aspects of high-quality research such as design, statistical analysis and data interpretation. Efforts such as the ASCO Journals Editorial Fellowship provide valuable access to research training [16]. The investment in more readily accessible research training that makes use of online modules and virtual mentorship may help address this gap.

Respondents from all countries overall reported lower levels of preparedness for practice in tasks such as leadership and effective management of oncology practice, and identification of gaps in the cancer delivery system. The global medical oncology curriculum outlined by the ESMO/ASCO Global Curriculum Working Group emphasised that service commitment should not compromise leadership and/or communication skills training [17]. System thinking and leadership training need to be actively incorporated in training curricula and instructional design. Innovative delivery methods making use of online learning and quality assurance projects during training can help address this gap in LMICs, UMICs and HICs.

Respondents identified high service commitment as a challenge faced during training. Skewed service to education ratio is a particular challenge with increasing work volume interfering with educational attainment. Other challenges for oncology training identified by respondents include the shortage of teaching faculty. A global effort is needed through investing in faculty development, South-South and North-South collaboration, sharing of curricula and educational resources, and harnessing the power of IT. Although we did not specifically gather data on the levels of burnout amongst oncologists, when asked to elaborate on challenges faced during their training, several respondents identified burnout and provider fatigue as a major concern. This is in keeping with several other studies conducted on burnout during training and during practice [18–20], and programs must incorporate measures to address this challenge.

Regarding career pathways, clinical oncology remains a viable option and the most common pathway in LMIC. Although this survey did not provide conclusive evidence of future directions, 75% of respondents from LMIC believe that the pathway should be separated into medical oncology and radiation oncology. The ongoing debate on the pros and cons of such separation, especially in LMIC will help to determine future directions and aid in deciding admission criteria, curriculum development and resource allocation for training. It is also interesting to note that, overall, 3% of respondents had no formal core oncology training and 2% reported training in a program with no formal accreditation. Some oncology training programs may have only local accreditation rather than regional or continental bodies. Furthermore, some regions particularly in LMICs, where there is a shortage of both oncologists and facilities, are using task shifting to train general practitioners who are not formally trained in oncology to deliver safe and effective cancer care [21].

There are several limitations to this study. As with any survey, respondents may not be representative of all providers in each system. We built off the same network of contacts similar to our previous survey study on oncology workload [10], and survey fatigue may have resulted in a lower response rate. As with our previous study, we were unable to attain responses from the USA or Russia as national associations in both these countries refused to participate in the study. As the survey was sent out via national associations in some countries, and relying on informal distribution in others, we were unable to determine the denominator (i.e. response rate). The strengths of our study include a high proportion of responses from LMICs, including trainees. As the future of the workforce in LMICs, and as an underrepresented population in the literature, it is imperative that these individuals are included in the discussion regarding curriculum development. The observation that a higher proportion of trainees from LMICs report self-funding for their training may be due to the fact that many travelled abroad for their training; however, due to low response rates for such questions, we were unable to analyse patterns for this movement. The content of this survey was related to training rather than workload, and as such, there may be a bias towards respondents who are particularly invested in medical education and training. Furthermore, only 5% of respondents provided radiation therapy only, and thus, these results may not be generalisable to radiation therapy training. Overall, 35% of respondents are now 15 years out of training which may impact the accuracy of their responses regarding the characteristics of their training program and/or their training program may have changed since. It should also be noted that the responses regarding preparedness for practice include responses from current trainees, which may have skewed the results towards lower preparedness for practice.

## Conclusions

In conclusion, our survey demonstrated that several challenges in oncology training are universal, such as inadequate preparedness for practice in leadership and effective management of oncology practice, identification of gaps in the cancer delivery system and burnout during training. Deficiencies in research output and dissemination are a particular challenge during training in LMICs. Innovative and sustained efforts are needed to address this. The investment in training in particular by the public sector would be vital to developing an effective physician workforce and decreasing the prevalence of self-funding in LMIC. Finally, further research is needed to elucidate the characteristics, quality and relevance of the global oncology workforce training, ideally by evaluating current efforts and generating knowledge to improve training. Such research is severely lagging behind biomedical and health services research in oncology. A concerted effort from the global oncology community is needed to address the major deficiency and to help position research into the training of the oncology workforce as one of cancer research’s priorities.

## Conflicts of interest

The authors declare that they have no conflicts of interest.

## Disclaimers

None.

## Funding

None.

## Presentations

This study has been presented at the 2019 ASCO Annual Meeting in Chicago, IL, USA and at the 2019 International Conference on Residency Education (ICRE) in Ottawa, ON, Canada and at the 2019 African Organization for Research and Training Conference in Maputo, Moazambique.

## Figures and Tables

**Figure 1.**
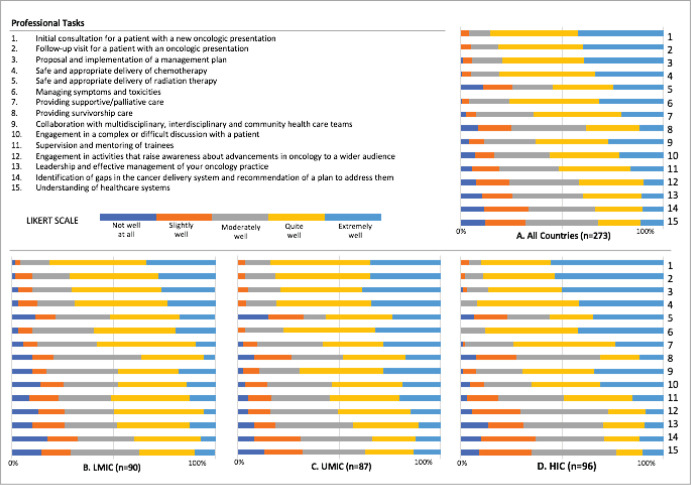
Preparedness for practice of oncologists from A. all countries, B. LMIC, C. UMIC, D. HIC based on their training in 15 professional tasks on a 5-point Likert scale (1 = not well at all, 2 = slightly well, 3 = moderately well, 4 = quite well, 5 = extremely well)

**Table 1.** Characteristics of study participants and their current practice.

Characteristic	LMIC (*n* = 90)	UMIC (*n* = 87)	HIC (*n* = 96)	All (*n* = 273)	*p*
Female (%)	37 (42)	33 (38)	55 (58)	125 (46)	0.015
Median age (years)	35	40	40	40	0.003
Trainees (%)	45 (51)	37 (42)	28 (19)	110 (40)	0.013
Year 1–3	31	15	12	58	
Year 4 or higher	14	22	16	52	
Practising physicians (%)	44 (49)	50 (58)	68 (71)	162 (60)	
Number of years since training					0.001
<5	17 (39)	8 (16)	18 (27)	43 (27)	
5–10	11 (26)	9 (18)	15 (22)	35 (22)	
11–15	2 (5)	17 (35)	6 (9)	25 (16)	
>15	13 (30)	15 (31)	28 (42)	56 (35)	
Therapy provided (%)					< 0.001
Systemic therapy	52 (58)	69 (79)	74 (77)	195 (71)	
Radiation therapy	0 (0)	6 (7)	8 (8)	14 (5)	
Both	38 (42)	12 (14)	14 (15)	64 (23)	
Speciality (%)					< 0.001
Medical oncologist	43 (48)	64 (74)	75 (78)	182 (67)	
Radiation oncologist	10 (11)	7 (8)	10 (10)	27 (10)	
Clinical oncologist	28 (31)	12 (14)	9 (9)	49 (18)	
Haematologist	4 (4)	1 (1)	0 (0)	5 (2)	
Paediatric oncologist	1 (1)	3 (3)	0 (0)	4 (2)	
Other	4 (4)	0 (0)	2 (2)	6 (2)	
Type of cancer					< 0.001
Solid	36 (40)	61 (70)	84 (88)	181 (66)	
Haematological	5 (6)	0 (0)	3 (3)	8 (3)	
Both	48 (54)	26 (30)	9 (9)	83 (31)	
Clinical practice setting					
System					< 0.001
Public	58 (65)	38 (44)	75 (80)	171 (63)	
Private	14 (16)	15 (17)	5 (5)	34 (13)	
Both	17 (19)	33 (38)	15 (16)	65 (24)	
Setting					0.033
Urban	78 (88)	85 (98)	88 (94)	251 (93)	
Rural	0 (0)	1 (1)	1 (1)	2 (< 1)	
Both	11 (12)	1 (1)	5 (5)	17 (6)	
Setting					0.004
Academic university	34 (38)	37 (43)	54 (56)	125 (46)	
Public or non-academic hospital	9 (10)	17 (20)	9 (9)	35 (13)	
Private hospital/clinic	7 (8)	13 (15)	5 (5)	25 (9)	
Cancer centre	39 (44)	20 (23)	28 (30)	87 (32)	

**Table 2.** Characteristics of core oncology training programs.

Characteristic	LMIC (*n* = 73–90)	UMIC (*n* = 77–87)	HIC (*n* = 89–96)	All (*n* = 239–273)	*p*
Median duration of core oncology training (years)	3	4	4	4	0.218
Core oncology training speciality (%)					< 0.001
Medical oncology	35 (47)	59 (78)	67 (75)	161 (67)	
Radiation oncology	3 (4)	5 (7)	6 (7)	14 (6)	
Clinical oncology	31 (41)	7 (9)	13 (15)	51 (21)	
None	4 (5)	2 (3)	2 (2)	8 (3)	
Other	2 (3)	3 (4)	1 (1)	6 (3)	
Median number of trainees in program	7	8	6	6	0.321
Setting (%)					
Hospital-based	47 (52)	46 (53)	59 (62)	152 (56)	0.366
University-affiliated	42 (47)	51 (59)	62 (65)	155 (57)	0.044
Other	5 (6)	5 (6)	3 (3)	13 (5)	0.645
Program accreditation (%)					0.039
Local	6 (8)	3 (4)	2 (2)	11 (5)	
National	37 (51)	53 (69)	65 (73)	155 (65)	
International	25 (34)	15 (20)	19 (20)	59 (25)	
None	0 (0)	3 (4)	1 (1)	4 (2)	
Unknown	5 (7)	3 (4)	2 (2)	10 (4)	
Funding source (%)					
Hospital	24 (33)	32 (42)	38 (43)	94 (39)	0.395
University	12 (16)	27 (35)	37 (42)	76 (32)	0.002
Self-funded	27 (37)	10 (13)	10 (11)	47 (20)	< 0.001
Host country	9 (12)	7 (9)	3 (3)	19 (8)	0.100
International organization	5 (7)	4 (5)	1 (1)	10 (4)	0.168
Other	10 (14)	6 (8)	18 (20)	34 (14)	0.072
Volume of patients					0.008
Not enough	5 (7)	2 (3)	3 (3)	10 (4)	
Adequate	39 (54)	43 (56)	68 (78)	150 (63)	
Too many	28 (39)	32 (41)	17 (19)	77 (33)	
Scholarly activities					
Regular assessments	62 (89)	41 (55)	70 (80)	173 (74)	< 0.001
Research	65 (89)	66 (86)	72 (80)	203 (85)	0.267
Abstract	24 (37)	19 (27)	37 (51)	79 (39)	0.014
Poster	13 (20)	28 (42)	42 (58)	83 (41)	< 0.001
Oral presentation	20 (31)	16 (24)	24 (33)	60 (30)	0.448
Publication	24 (37)	32 (48)	43 (60)	99 (49)	0.029
Other	12 (19)	5 (8)	11 (15)	28 (14)	0.176
Tumour boards	70 (96)	67 (89)	86 (96)	223 (94)	0.170
Site-specific	54 (76)	54 (76)	80 (93)	188 (83)	0.005
Journal clubs/grand rounds/ didactic teaching sessions	67 (92)	69 (90)	82 (91)	218 (91)	0.893

**Table 3.** Preparedness for practice of oncologists from LMICs, UMICs and HICs based on their training in fifteen professional tasks on a 5-point Likert scale (1 = not well at all, 2 = slightly well, 3 = moderately well, 4 = quite well, 5 = extremely well).

Professional task	LMIC (*n* = 90)	UMIC (*n* = 87)	HIC (*n* = 96)	All (*n* = 273)	p**
	Mean score*				
1. Initial consultation for a patient with a new oncologic presentation	4.1	4.2	4.4	4.2	0.009
2. Follow-up visit for a patient with an oncologic presentation	3.9	4.1	4.4	4.2	0.001
3. Proposal and implementation of management plan for a patient with an oncologic presentation	3.9	4.1	4.3	4.1	0.004
4. Safe and appropriate delivery of systemic therapy	3.8	4.1	4.3	4.1	0.002
5. Safe and appropriate delivery of radiation therapy	3.4	3.3	3.6	3.4	0.535
6. Managing symptoms and toxicities	3.7	4.1	4.3	4.0	<0.001
7. Providing supportive/palliative care	3.5	3.7	3.9	3.7	0.023
8. Providing survivorship care	3.1	3.3	3.1	3.2	0.301
9. Collaboration with multidisciplinary, interdisciplinary and community health care teams to provide optimal patient care	3.4	3.8	3.9	3.7	0.006
10. Engagement in a complex or difficult discussion with a patient	3.2	3.5	3.8	3.5	0.010
11. Supervision and mentoring of trainees	3.3	3.5	3.4	3.4	0.562
12. Engagement in activities that raise awareness about advancements in oncology to a wider audience	3.2	3.4	3.0	3.2	0.017
13. Leadership and effective management of your oncology practice	3.3	3.3	2.9	3.1	0.086
14. Identification of gaps in the cancer delivery system and recommendation of a plan to address them	3.0	3.1	2.9	3.0	0.726
15. Understanding of healthcare systems	3.0	3.0	2.9	3.0	0.466

* Although the ordinal data were not normally distributed, the medians were often identical between groups. The means are presented to highlight subtle differences.

** *p*-value from Kruskal–Wallis test.
